# Epithelial and macrophage cell interaction in cervical cancer through single-cell RNA-sequencing and spatial analysis

**DOI:** 10.3389/fimmu.2025.1537785

**Published:** 2025-04-09

**Authors:** Zhichao Wang, Long Cheng, Guanghui Li, Huiyan Cheng

**Affiliations:** ^1^ Department of Pediatric Surgery, First Hospital of Jilin University, Changchun, Jilin, China; ^2^ Department of Intensive Care Unit, First Hospital of Jilin University, Changchun, Jilin, China; ^3^ Department of Vascular Surgery, First Hospital of Jilin University, Changchun, Jilin, China; ^4^ Department of Obstetrics and Gynecology, First Hospital of Jilin University, Changchun, Jilin, China

**Keywords:** cervical cancer (CC), tumor microenvironment (TME), macrophages, immunotherapy, cell-cell communication

## Abstract

**Background:**

Cervical cancer (CC) is a major global health issue, ranking sixth in cancer-related mortality. The tumor microenvironment (TME) plays a crucial role in tumor growth. This study explored the cellular composition and immunological landscape of CC using various genomic data sources.

**Methods:**

Data from the Cancer Genome Atlas and Gene Expression Omnibus were analyzed, including single-cell RNA sequencing, spatial transcriptome analysis, and survival data. Gene set variation analysis (GSVA) identified pathways in CD8+ cells, macrophages, and epithelial cells. Immunohistochemistry assessed marker expression in CC and normal tissues. Tumor immune dysfunction and exclusion (TIDE) scores differentiated high- and low-macrophage groups. Cell–cell communication analyses highlighted interactions between macrophages and epithelial cells.

**Results:**

Macrophage markers correlated with overall survival (OS) and disease-free survival (DFS). Epithelial cell subgroups 1 and 4, along with CD8+ T cells, were associated with OS. TIDE scores varied between groups. Specific ligand-receptor interactions were found between macrophages and epithelial cell subgroup 1. Triptolide was effective in epithelial cell subgroup 1, while memantine was more effective in macrophages.

**Conclusion:**

Epithelial-macrophage interactions in the TME are crucial for CC progression and treatment, offering a potential immunotherapeutic strategy.

## Highlights

- Analysis integrating three cervical cancer datasets, supplemented with IHC experiments for validation.
- Comprehensive perspective on the cervical cancer TME provided through single-cell RNA sequencing (scRNA-seq) and extensive transcriptomic analysis.
- Identification of potential drug targets and biomarkers for personalized therapeutic strategies in cervical cancer.

## Introduction

Cervical cancer (CC) ranks as the fourth most common female malignancy, with an estimated 604,000 new cases and 342,000 deaths worldwide annually (1). While vaccination and screening programs have contributed to reducing the incidence of CC in developed countries, it remains a significant cause of morbidity and mortality among women in some low- and middle- income nations (2). Most CCs are primarily caused by persistent human papillomavirus (HPV) infection (3).

The treatment of CC depends on various factors, including the stage of cancer, whether it has spread to other parts of the body, the size of the tumor, the patient’s age, and overall health. According to guidelines from the National Comprehensive Cancer Network Center, primary treatment modalities include surgery, radiation, and chemotherapy alone or in combination (4). Some patients with early or locally advanced CC may achieve a certain degree of remission with a higher survival rate through radical resection or concurrent radiotherapy (5). However, the prognosis and treatment outcomes for patients with refractory CC, including those with recurrent, persistent, or metastatic CC, remain unsatisfactory (6–8).

Complex and dynamic interactions between epithelial cells and macrophages are a hallmark of the tumor microenvironment (TME). These interactions play a pivotal role in tumor initiation, progression, and metastasis. Epithelial cells, which constitute the major cellular component of tumors, actively influence macrophage function through the secretion of cytokines and the modulation of macrophage phenotypes. Conversely, macrophages reciprocally regulate epithelial cell proliferation, migration, and invasion by releasing immunomodulatory factors. This bidirectional crosstalk is critically involved in tumor immune evasion, immune clearance, and the development of drug resistance. Within the TME, macrophages exhibit remarkable plasticity and can adopt diverse phenotypes. While tumor-associated macrophages (TAMs) often promote tumor progression, certain macrophage subsets can exert anti-tumor effects (9). Consequently, a deeper understanding of the intricate interplay between epithelial cells and macrophages is essential for identifying novel therapeutic targets and advancing the development of effective immunotherapeutic strategies.

The remarkable efficacy of immunotherapy in CC highlights the significance of immunotherapy interventions targeting angiogenesis in the tumor microenvironment (TME) (10, 11). Therefore, pembrolizumab combined with chemotherapy (with or without bevacizumab) has become the first-line treatment choice for the programmed cell death ligand 1 positive patients with metastatic, persistent, and recurrent CC (7). However, some targeted therapies may be non-responsive due to immune infiltration of tumor cells (10). Therefore, elucidating the role of TME in the pathogenesis and targeted therapy of CC is imperative.

In CC, persistent infection with high-risk human papillomavirus (HPV) is widely recognized as the primary driver of carcinogenesis. However, emerging evidence suggests that cellular mutations within the TME, particularly in epithelial cells and immune cells such as macrophages, play a critical role in tumor progression and therapeutic response (12). For instance, phenotypic alterations and genetic mutations in macrophages can reshape the TME, thereby influencing tumor immune evasion and survival. Similarly, genetic mutations in epithelial cells have been implicated in regulating their proliferative capacity, invasive potential, and sensitivity to immunotherapeutic interventions (13). These studies demonstrate that mutations not only dictate the biological behavior of tumor cells but also modulate macrophage function within the TME, ultimately impacting patient outcomes. These collective findings underscore the significance of investigating epithelial and macrophage mutations in CC, as such insights could provide a mechanistic understanding of tumor evolution and inform the development of novel therapeutic strategies.

In this study, we explored the functions, subpopulations, mutations, and characteristics related to patient survival and treatment of epithelial cells and macrophages within tumors, using multiple analytical methods and datasets to evaluate the cell types and their interactions within the TME. Moreover, we compared the expressions of various genes in single-cell data and analyzed their differential expression. Additionally, we identified the functional differences revealing specific biological characteristics of the various cell types. We conducted subpopulation analysis to classify the epithelial cells into distinct categories to understand their diversity and functional differentiation. Correlation analysis with patient survival revealed associations between epithelial cell subsets and macrophages, impacting patient prognosis. The immunotherapy analysis for high- and low-risk groups provided valuable insights into the application prospect and its efficacy in different risk groups. Mutations in epithelial cells and macrophages were examined to comprehend their role in tumors, offering clues for further study of tumor development. By analyzing spatial transcriptome data, intercellular communication patterns, and drug sensitivity between epithelial cells and macrophages, we revealed their interactions and responses to therapy, thus guiding future research endeavors and therapeutic strategies.

## Methods

### Dataset

We used The TCGA-CESC dataset was downloaded using the TCGAbiolinks package (14) from The Cancer Genome Atlas (TCGA) (https://portal.gdc.cancer.gov/) and analyzed as the test set. After excluding samples without clinical information, sequencing data from 304 CC samples with prognostic OS clinical information were obtained. The sequencing data for CC (CESC) were normalized to fragments per kilobase pair million format. Corresponding clinical data were obtained from the UCSC (University of California, Santa Cruz) Xena database (http://genome.ucsc.edu) (15). Additionally, the CC-related dataset GSE44001 (16) was acquired from the Gene Expression Omnibus (GEO) database using the R package GEO query (17). These samples were from *Homo sapiens*, and the chip platform was GPL14951, containing 300 CC samples with clinical information regarding prognosis and DFS. Furthermore, the CC single-cell dataset GSE168652 (18) and CC cell space transcriptome dataset GSE208654 (19) were downloaded. The GSE168652 samples were from *Homo sapiens*, with the chip platform being GPL24676, consisting of one CC sample and one healthy sample. The GSE208654 dataset comprised normal tissues, precancerous lesions, and CC tissue samples (18) (17) (16), (15) (15) (Li et al., 2021)(Li et al., 2021)(Li et al., 2021)(Li et al., 2021)(Li et al., 2021)(Li et al., 2021)(Li et al., 2021) (15) (Li, Guo et al., 2021) (15) with samples from *Homo sapiens* and the chip platform being GPL24676. These datasets were further analyzed. The specific information is shown in **Supplementary Table S1**.

### Single-cell analysis

The “CreateSeuratObject” function of the R package Seurat v4.0 (20) was used to import the counts matrix of all samples in the scRNA-seq dataset GSE168652, creating a Seurat object. The parameters were set to include genes expressed in at least three cell types and at least 200 genes expressed in each cell. The proportion of mitochondrial genes indicates whether the cells are in a steady state. Typically, a cell might be under stress when it has a higher proportion of mitochondrial genes than all other genes. Therefore, we filtered out cells with >20% mitochondrial gene content. Low-quality cells or empty droplets typically have fewer genes; hence, we filtered out cells with features under 250.

Subsequently, we normalized the sequencing depth of the scRNA-seq dataset GSE168652 using the “SCTransform” function. Principal component analysis (PCA) was then applied to identify the principal components (PCs), which were visualized using the “Elbowplot” function to determine the *p*-value distribution. Finally, 10 PCs were selected for unified manifold approximation, projection (tSNE) analysis, and dimension reduction. The “FindNeighbors” function was used with default parameters and the 10 PC dimension parameters to construct the k-nearest neighbors based on Euclidean distance in the base PCA space. By calling the “FindClusters” function, the “clustree” function was applied to find a resolution of 0.5 to divide the cells into different clusters. Finally, the “RunTSNE” function dimension was used for dimensionality reduction and visualization of the dataset.

### Cell type annotation and single-cell taxa differential genes

Using “SingleR (21), the “DotPlot” function was used to display the expression levels of model genes in different cell types. To identify differentially expressed genes among cell clusters, we used the “FindAllMarkers” function to compare the gene expression in a cell to that in all other cells using the Wilcoxon rank-sum test. Differential genes with logFC >1 and adjusted *p*-value < 0.05 for each cell cluster were retained as cell marker genes for further study.

### GSVA of different cell subsets

Gene Set Variation Analysis GSVA (22) was applied to evaluate the pathways enriched in different samples. The human hallmark gene set was obtained using the R package msigdbr, and GSVA was performed on all genes in the single-cell dataset to calculate the functional enrichment differences among various cell subsets. The screening criterion for GSVA was adjusted *p* < 0.05.

### Functional and pathway enrichment analyses

GO (23) analysis is a common method for large-scale functional enrichment studies, including BP, MF, and CC. KEGG (24) is an extensively used database storing information on genomes, biological pathways, diseases, and drugs. The R package clusterProfiler (25) was applied for GO and KEGG annotation analyses of differentially expressed genes. The entry screening criteria, adjusted *p*-value of under 0.05 and false-discovery rate value (*q*-value) of less than 0.25, were considered statistically significant. The Benjamini–Hochberg (BH) method was used *p*-value correction.

### Survival analysis

Single-sample GSEA (ssGSEA) quantified the abundance of each gene in a dataset sample. We used the R package GSVA to calculate the cell correlation score of each sample in the TCGA and GSE44001 datasets based on the expression matrix of each sample. We integrated the TCGA-CESC dataset for overall survival (OS) and disease-free survival (DFS) prognosis, the GSE44001 dataset, and the TCGA-CESC dataset to categorize cell grades and construct Kaplan-Meier (KM) survival curves. This analysis facilitated the optimal stratification of cell groups. Subsequently, based on the most favorable stratification, cervical cancer (CC) patients within the TCGA-CESC dataset were segregated into high and low groups.According to the best grouping, CC patients in the TCGA-CESC dataset were divided into high and low groups.

### Somatic mutation analysis of CC subtypes

“Masked Somatic Mutation” data of CESC samples were selected through the TCGA platform and preprocessed using VarScan software and the R package maftools (26).

### Immune-related analysis

The TIDE (27) (http://tide.dfci.harvard.edu) score predicts the potential response to immune checkpoint blockade therapy. The TIDE algorithm models two main mechanisms of tumor immune evasion: induction of T cell dysfunction in tumors with high cytotoxic T lymphocyte (CTL) infiltration and prevention of T cell infiltration in tumors with low CTL levels. The expression of each gene in tumor patients was assessed based on its interaction with CTL infiltration level to influence the survival rate in a large tumor group with T cell dysfunction. Based on the TIDE analysis results, we calculated and compared the differences in TIDE grouping between the high and low groups in the TCGA-CESC dataset using the Mann–Whitney U test or Wilcoxon rank-sum test. The test was also applied to calculate the differences in tumor mutation load between the high- and low-TMB groups using TCGA data obtained from CESG. A *p*-value of less than 0.05 was considered statistically significant.

### Space transcriptome analysis

Samples from the single-cell spatial transcriptome dataset GSE208654 were imported using the “Load10X_Spatial” function of the R package Seurat v4.0 (20) and created as Seurat objects. The GSE208654 dataset was normalized using the “SCTransform” function. PCA was applied to identify significant PCs, and ten PCs were selected for Uniform Manifold Approximation and Projection analysis for dimensionality reduction. The “FindNeighbors” default parameter and ten PC dimension parameters were used to construct the k-nearest neighbors based on Euclidean distance in the base PCA space. The cells were then divided into different clusters using the “FindClusters” function. The Human Primary Cell Atlas Data dataset was applied for cell annotation via the singleR function, combined with the previously identified epithelial marker genes in cells. Subsequently, the “FindTransferAnchors” and “TransferData” functions were used to combine single-cell spatial data, followed by visualization of gene expression in different cell types using the “SpatialDimPlot” function.

### Cell communication analysis

Multicellular organisms communicate via cytokines and membrane proteins to regulate their life activities. Among these, receptor-ligand-mediated intercellular communication is essential for various BPs, such as development, cell differentiation, and disease. Moreover, cell communication analysis deduces the interaction among cells by measuring the expression and pairing of receptors and ligands in various cell types.

To increase confidence in potential ligand-receptor interactions of cell-to-cell communication, we adopted a strategy based on consensus analysis of multiple approaches and compared the results of different ligand-receptor inference methods to predict interactions. We used the “liana_wrap” and “liana_aggregate” functions in the R package liana for cell communication analysis, which runs other methods in the background and generates a consensus. This approach could be beneficial in identifying ligand-receptor interactions that are highly significant in different methods, including CellPhoneDB. These methods include the following:

Connectome: a network analysis-based approach inferring functional connections between cells from single-cell transcriptome data. Log2FC: a method based on gene expression differences identifying ligand-receptor pairs that change under different conditions from single-cell transcriptome data. NATMI: a machine learning-based method predicting cell-to-cell ligand-receptor interactions from single-cell transcriptome data. SingleCellSignalR: a pathway-based approach reconstructing cell-to-cell signaling networks from single-cell transcriptome data. CellChat: a probabilistic graph model-based approach inferring cell-to-cell communication patterns from single-cell transcriptome data.

### Single-cell susceptibility analysis

Beyondcell (28) is a single-cell drug sensitivity analysis method that identifies subsets of tumor cells with different drug responses and proposes cancer-specific therapies. In this study, we used the R package Beyondcell for single-cell drug sensitivity analysis as follows: First, the single-cell drug sensitivity was determined by the expression of drug-related genes in each single cell. Subsequently, single cells with similar drug sensitivity were grouped using a clustering algorithm to form therapeutic clusters (TCs). Finally, differences in drug sensitivity among TCs were analyzed to guide sensitivity-based selection. Beyondcell has been validated in five single-cell datasets and has demonstrated that TCs could be used to target malignant cells in cancer cell lines and tumor patients.

### AUCell

AUCell identifies cells with active gene sets in single-cell RNA sequence data and analyzes whether the input gene set is enriched in the genes expressed in each cell, based on the AUC. The distribution of AUC scores facilitates the exploration of the relative expression of features. Given that the scoring method employed by AUCell relies on ranking, the tool functions through assessing gene expression following established standard protocols. Furthermore, because cells were evaluated individually, AUCell could be applied to larger datasets and expression matrices as needed. Therefore, we selected cell marker genes for AUCell scoring to identify cell populations with high scores.

### Immunohistochemistry

Immunohistochemistry (IHC was conducted as described previously (29). The protein levels of three macrophage-related genes were analyzed by IHC using tissue microarrays from incisional biopsy specimens taken before treatment and this study was approved by the Ethical Review Board for Research; All the antibodies were purchased from Zhongshan Goldbridge Biotechnology for immunohistochemistry staining. Each tumor was represented by a tissue core on a microarray. The tissue samples were fixed in 10% neutral-buffered formalin and embedded in paraffin before slicing into 4-mm-thick sections and mounting on glass slides. Antigen retrieval was performed to enhance antigenicity, and the sections were blocked to minimize nonspecific binding. Subsequently, the sections were incubated overnight at 4°C with primary antibodies targeting CD163, CD14, and CD68, respectively, followed by HRP-coupled anti-rabbit (Pasilla) or anti-mouse (tubulin) secondary antibody. Finally, the slides were treated with a chromogenic substrate to visualize the antigen-antibody interaction, which was enhanced by hematoxylin counterstaining. The stained sections were examined under an optical microscope to assess CD163, CD14, and CD68 expression levels, and the intensities were compared between the tumor and adjacent groups to determine the differential expression patterns using ImageJ software.

### Statistical analysis

All data were processed and analyzed using R software, version 4.2.0. Continuous variables were presented as mean ± standard deviation, and the Wilcoxon rank-sum test was used for comparison between the two groups. Correlation coefficients between different molecules were calculated using Spearman’s correlation analysis, unless otherwise specified. All results were considered statistically significant at an adjusted *p-*value <0.05.

## Results

Flow diagram of single cell spatial transcriptome (**Supplementary Figure S1**).

### Cell type annotation

**Figure 1:**
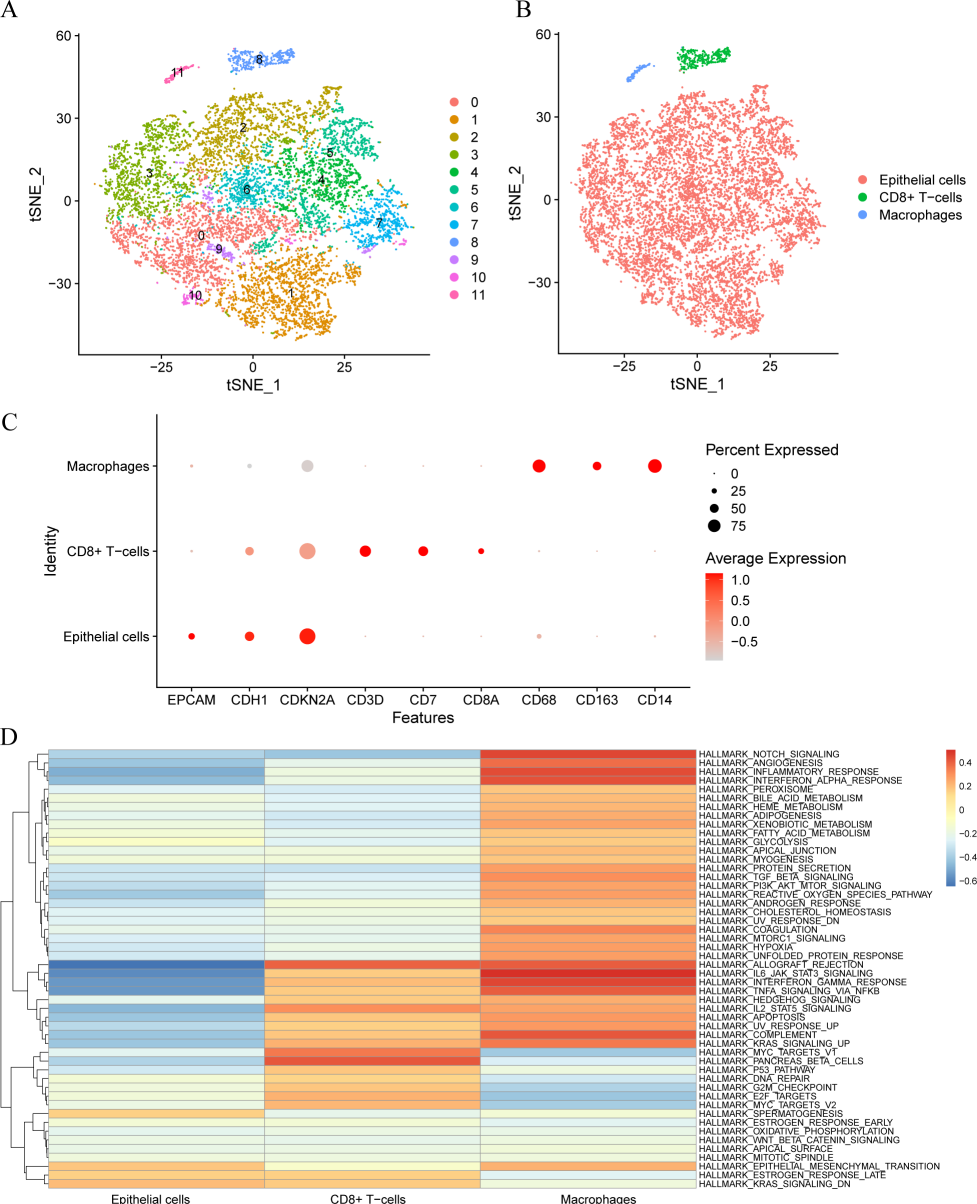
Cell type annotation. **(A)**. Clustering into 12 cell clusters by tSNE. **(B)**. The Cells were annotated into 3 cell types by R package singleR: Epithelial Cells, CD8+T Cells, and Macrophages. **(C)**. Visualization of bubble plots of expression levels of nine Model Genes. The deeper the color according to the higher expression level, the bigger the circle, said gene expression within the cells of the higher proportion. **(D)**. Gene set variation analysis (GSVA) between different cells.

The cells were classified into 12 clusters based on the single-cell dataset GSE168652 using t-distributed stochastic neighbor embedding (tSNE) for visual dimension reduction at a resolution of 0.5. The tSNE diagram illustrates distinct groups of cells (**Figure 1A**). Subsequently, the R package SingleR was used to categorize the cell clusters into three cell types (**Figure 1B**): macrophages, CD8+ T cells, and epithelial cells. The expressions of nine model genes including EPCAM, CDH1, CDKN2A, CD3D, CD7, CD8A, CD68, CD163 and CD14 in the single-cell dataset were displayed on a bubble plot (**Figure 1C**). The gene sets underlying the differences in the three cell types were identified using gene set variation analysis (GSVA). The results indicated that most HALLMARK gene sets have high scores in macrophages and epithelial cells, while most cells had low scores (**Figure 1D**).

### Gene set enrichment analysis

By GO and KEGG pathway enrichment analyses, we explored the biological processes (BPs), cellular components (CCs), and molecular functions (MFs) associated with various cell marker genes.

GO analysis revealed that epithelial cells regulate endopeptidase and peptidase inhibitor activity, as well as calcium-dependent protein binding, suggesting their involvement as structural constituents of the cytoskeleton. CD8+ T cells and major histocompatibility complex (MHC) class II are involved in protein complex binding, MHC-protein complex interaction, and ATP-dependent protein folding chaperone activities, with implications in chemokine activity and unfolded protein responses.

Macrophages are involved in peptide-MHC class II receptor activity, immune receptor activity, and MHC class II protein complex interactions, indicating their role in antigen presenting immune response modulation (**Supplementary Figure S2A**). The KEGG pathway analysis revealed that epithelial cells are associated with pathways such as amoebiasis, interleukin (IL)-17 signaling, p53 signaling, estrogen signaling, and *Staphylococcus aureus* infection. CD8+ T cells are linked to pathways including rheumatoid arthritis, viral protein interaction with cytokines and cytokine receptors such as interferon gamma receptor, tumour necrosis factor alpha and so on, lipid metabolism, atherosclerosis, Chagas disease, measles, and antigen processing and presentation. Meanwhile, macrophages are involved in pathways such as leishmaniasis, phagosome formation, rheumatoid arthritis, viral protein interaction with cytokines and cytokine receptors, lipid metabolism, atherosclerosis, and Chagas disease (**Supplementary Figure S2B**).

Additionally, subgroup analysis revealed that epithelial cells can be divided into five categories. Genes with a logfold-change (FC) >1 and adjusted *p* < 0.05 were identified as markers through KEGG analysis. The results indicated that Epithelial_1 is associated with complement and coagulation pathways, focal adhesion, AGE-RAGE (advanced glycation end products and the receptor for AGEs) signaling pathway in diabetic complications, and the HIF-1 signaling pathway; Epithelial_2 is involved in oocyte meiosis, cell cycle regulation, cellular senescence, the p53 signaling pathway, and apoptosis. Epithelial_4 mainly interacts with cytokine–cytokine receptors, Toll-like receptor signaling, chemokine signaling, and the RIG-1-like receptor signaling pathway (**Supplementary Figures S2C**).

### Survival analysis

**Figure 2:**
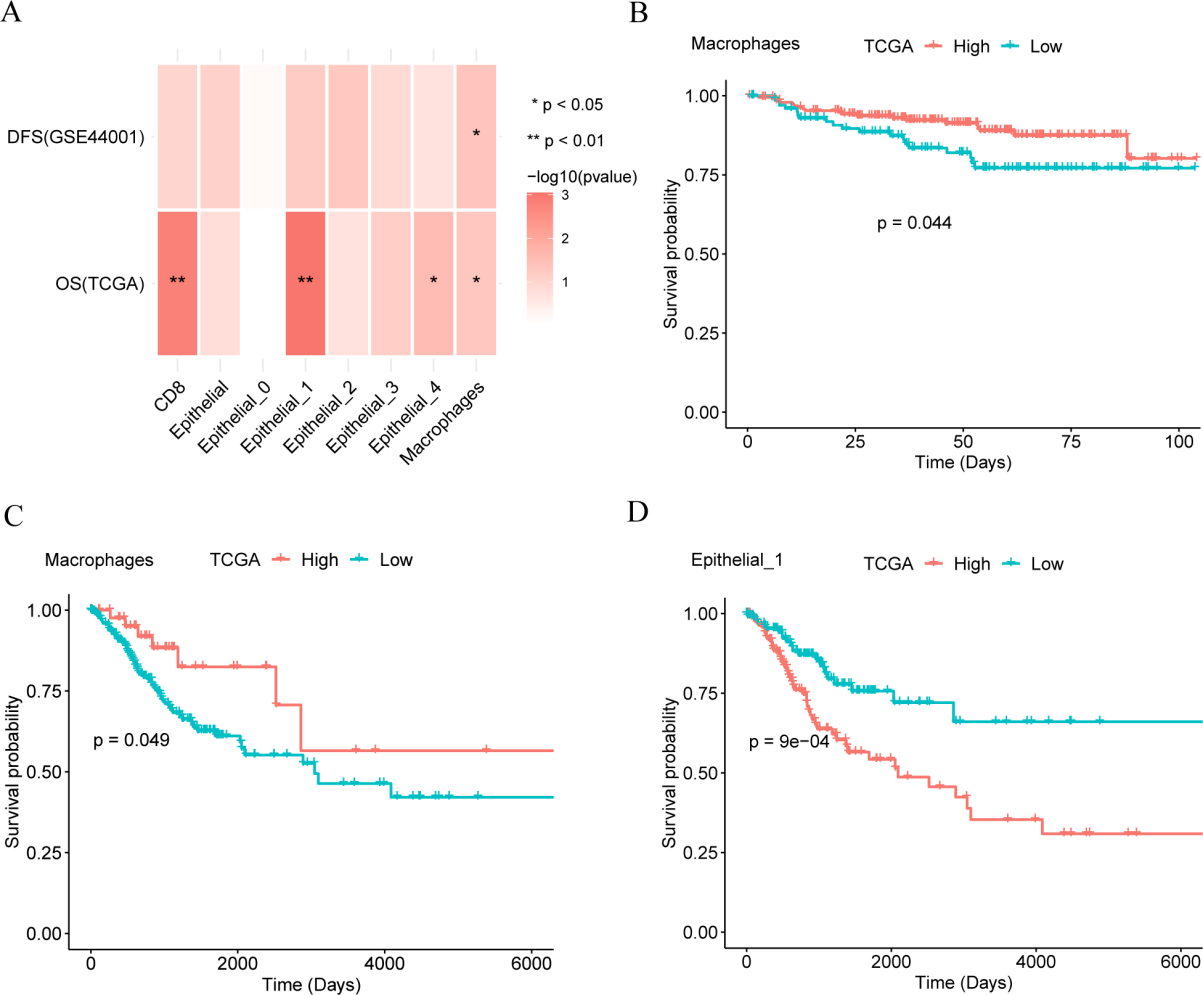
Survival analysis. **(A)**. The relationship between different cell marker genes and patients’ overall survival (OS) and disease-free survival (DFS). **(B, C)**. Kaplan-Meier (KM) curves of macrophage marker gene score and disease-free survival (DFS, panel **B**) and overall survival (OS, panel **C**) of patients. **(D)**. Kaplan-Meier (KM) curves of epithelial cell subset 1 and overall survival (OS).

To explore the antitumor effects of different cell marker genes in CC, we analyzed the correlation between overall survival (OS) in different cancer types and cell marker genes using Kaplan–Meier (KM) curve analysis. Correlation analysis (**Figure 2A**) revealed that macrophage marker genes were associated with OS and disease-free survival (DFS) in patients. Additionally, subpopulations 1 and 4 of epithelial cells, along with CD8+ T cells, showed correlations with patient OS. The KM curve showed that the macrophage marker gene group showed a statistically significant difference in DFS (**Figure 2B**) and OS (**Figure 2C**) between patients with high and low expression of macrophage marker genes (*p* < 0.05), where a higher score indicated better outcomes. Furthermore, a statistically significant correlation was observed between epithelial subpopulation 1 marker genes and OS (**Figure 2D**) (*p* < 0.001), where a higher score indicated a poorer prognosis. Macrophages were associated with both OS and DFS, while epithelial subpopulation 1, which predominates in tumor tissue, was primarily linked to OS.

### Immune correlation analysis

**Figure 3:**
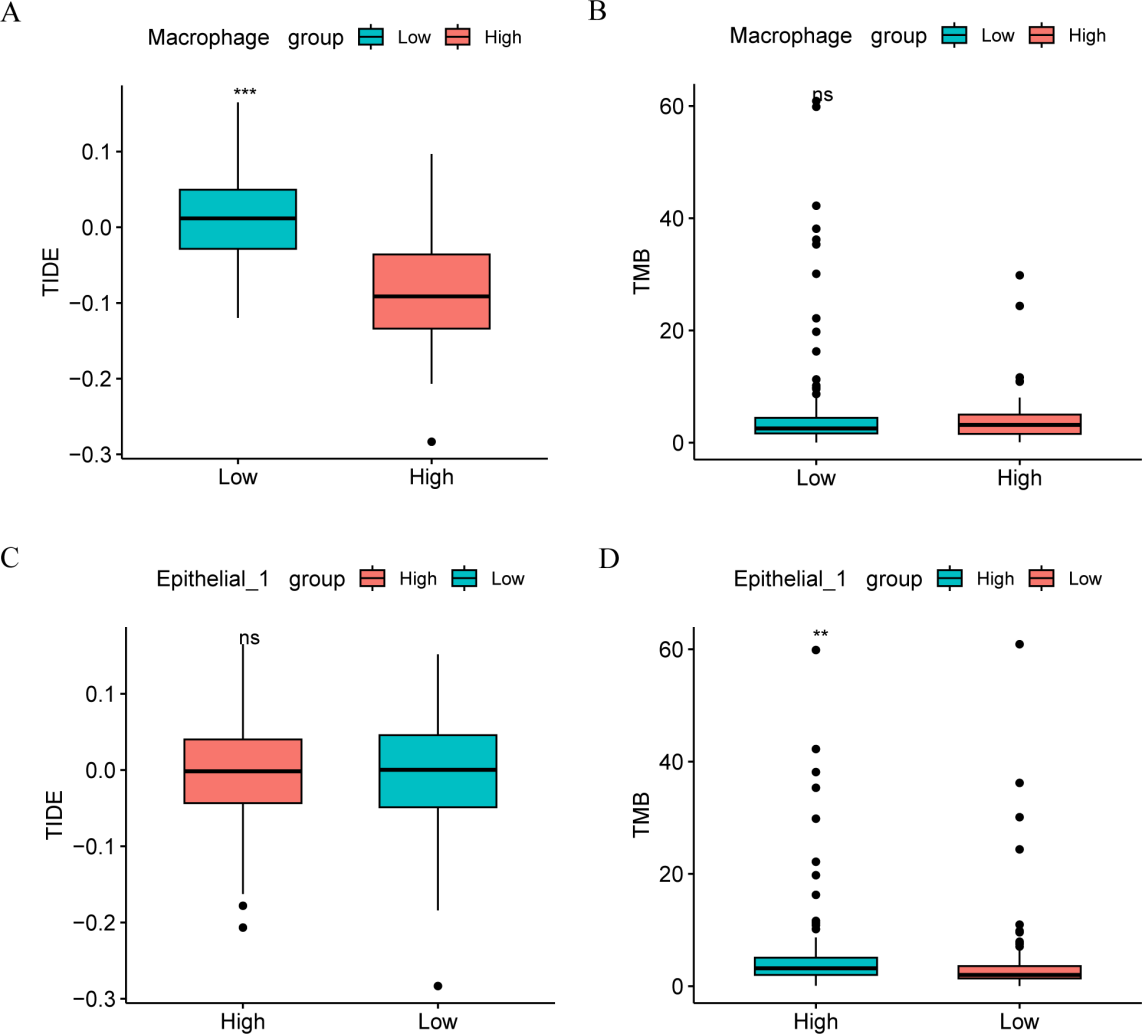
Immune correlation analysis. Comparison of TIDE immunoscore **(A)** and TMB score **(B)** groups between high and low macrophage groups in TCGA-CESC data set. Comparison of TIDE immune score **(C)** and TMB score **(D)** groups between macrophage high and low groups in TCGA-CESC data set. ns, *p* ≥ 0.05; *p < 0.05; **p < 0.01; ***p < 0.001.

Given the current important role of immunotherapy in tumors. The sensitivity of CC patients to immunotherapy in the high- and low-risk groups of the Cancer Genome Atlas cervical squamous cell carcinoma and endocervical adenocarcinoma (TCGA-CESC) dataset was assessed using the tumor immune dysfunction and exclusion (TIDE) algorithm, with results analyzed through the Wilcoxon rank-sum test. As shown in **Figure 3**, the TIDE immunotherapy score of CESC patients exhibited a significant difference between the high and low macrophage score groups (*p* < 0.001, **Figure 3A**), with the high-score group showing lower scores compared to the low-score group. Therefore, the high-score group may have a more favorable immunotherapy response than the low-score group. Analysis of TCGA-CC patient samples from the CESC dataset revealed no statistically significant difference in TMB within macrophages between the high- and low-score groups (*p* > 0.05) (**Figure 3B**).

In contrast to macrophages, TIDE immunotherapy scores in CESC patients did not significantly differ between the high- and low- score groups in Epithelial_1 group (**Figure 3C**). However, TMB in Epithelial_1 showed a significant difference between the high- and low-score groups (*p* < 0.01, **Figure 3D**).

### Somatic mutation analysis of high and low-macrophage groups

The analysis of the frequency of gene mutations in the high- and low-macrophage groups, conducted using the R package maftools, showed a higher mutation frequency in PIK3CA in macrophages between the high and low groups (**Supplementary Figure S3A**). Additionally, we analyzed the changes in biological function caused by mutations in high- and low-density groups of macrophages. The results demonstrated that the increased mutation frequency associated with functional changes in macrophages was concentrated in the RTK-RAS and Hippo signaling pathways (**Supplementary Figure S3B**), while the lesser mutation group was focused on the RTK-RAS and NOTCH signaling pathways (**Supplementary Figures S3C**).

Finally, based on the mutation and Drug Gene Interaction database (DGIdb), we integrated different cancer gene subtypes from patients into the medicinal database (Gene Druggability) to explore the interactions between drugs and genes. The results (**Supplementary Figures S3D, E**) revealed that in the macrophage high group, the predicted drugs were likely to target the druggable genome (*CACNA1H*, *CASP8*, *DMD*, *EP300*, and *HMCN1*). Conversely, for the macrophages in the low group, the potential effects of the drugs were detected on the druggable genome (*ADGRV1*, *DMD*, *DST*, *EP300*, and *MUC16*).

### Somatic mutation analysis of high and low groups in epithelial subpopulation 1

The R package maftools was used to analyze the frequency of gene mutations in the high and low groups of epithelial subpopulation 1. Similar to the mutation frequencies observed in the high-low subgroups of macrophages, both PIK3CA and TTN had high mutation frequencies in the high-low subgroups for epithelial subpopulation 1 (**Supplementary Figures S4A**). Additionally, we analyzed the changes in biological function caused by mutations in epithelial subpopulation 1 between the high and low subgroups. The results showed that mutations in the epithelial cells level 1 subgroup led to altered biological functions between the high and low subgroups, are both primarily concentrated in the RTK-RAS and NOTCH signaling pathways (**Supplementary Figures S4B, C**).

Finally, gene druggability and drug-gene interactions in different cancer subtypes were explored based on the mutation profiles and DGIdb. The results (**Supplementary Figures S4D, E**) indicated that the predicted drugs were likely to target the druggable genome (*ADGRV1*, *DMD*, *DST*, *EP300*, and *MUC16*) in the high subgroup of epithelial subpopulation 1. Conversely, in the low group, the drug prediction suggested potential effects on *ADGRV1*, *CREBBP*, *DMD*, *DST*, and *EP300*.

### Spatial transcriptome analysis

**Figure 4:**
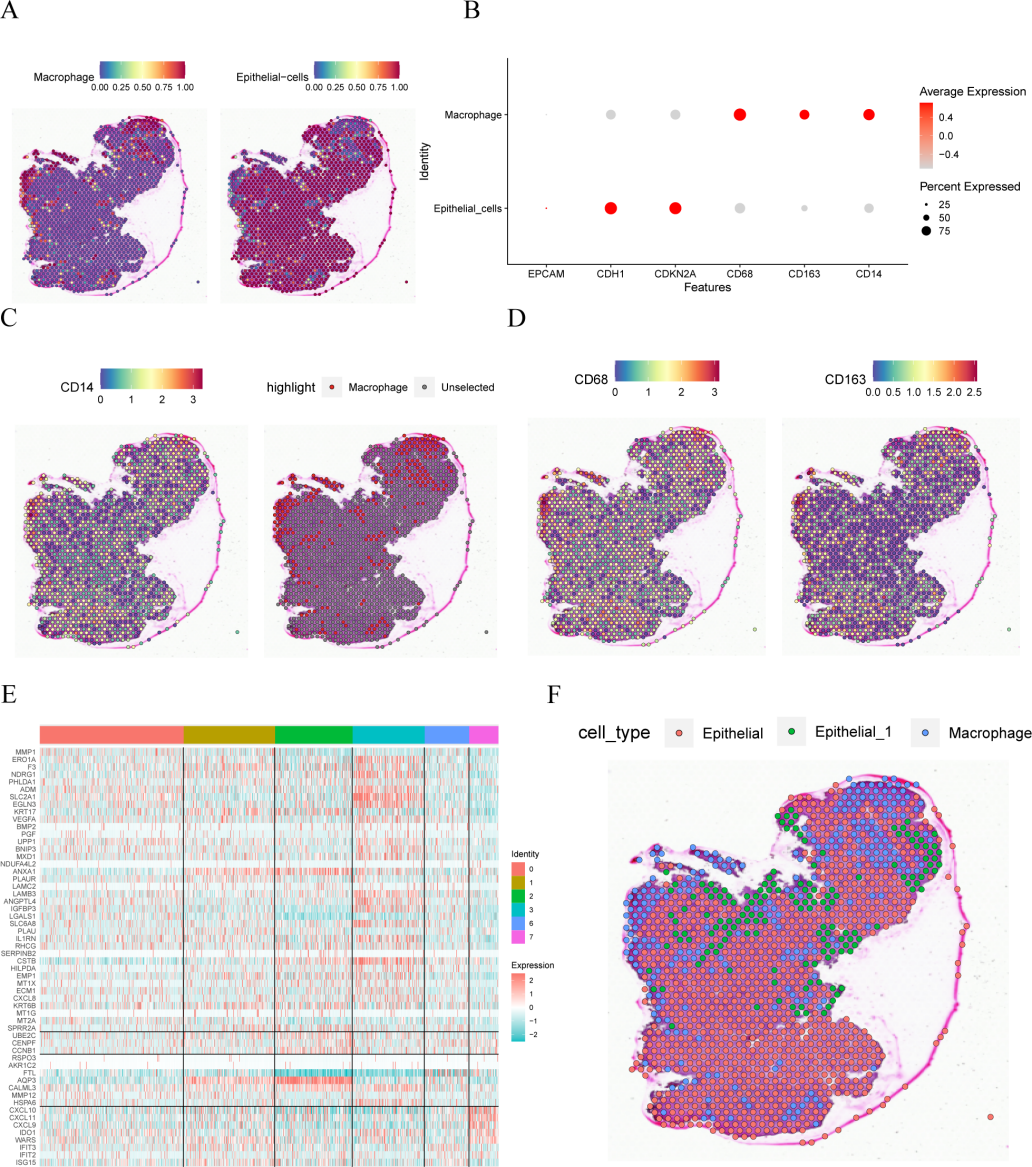
Spatial transcriptome analysis. **(A)**. spatial location of different cell comments. **(B)**. Visualization of bubble plots of expression levels of the six Model Genes. **(C, D)** Spatial location expression of CD14, CD68 and CD163 genes. **(E)**. Heat map of the expression of marker genes in different subsets of GSE168652 in the single cell dataset in the idle dataset. **(F)** Annotation of the spatial location of different cells.

We used the R package SingleR to identify two cell types in the spatial transcriptome dataset GSE208654: macrophages and epithelial cells, along with their spatial distribution (**Figure 4A**). The expressions of the six model genes in the single-cell dataset were represented in the bubble plot (**Figure 4B**), which exhibited similarities to those in the single-cell dataset GSE168652. Furthermore, **Figures 4C, D** shows the distribution of *CD14*, *CD68*, and *CD163* genes in the cells, primarily located within the macrophage region.

Upon examining the GSE168652 dataset, specific marker genes were identified for the ‘Epithelial_1’ single-cell subgroup. Concurrent analysis with the GSE208654 dataset revealed that the gene MMP-SPRR2A demonstrated notably increased expression within the ‘Epithelial_1’ population. Furthermore, **Figure 4E** illustrates the expression profiles of various marker genes across the single-cell landscape in the GSE168652 dataset. In alignment with the expression data, and using the annotation available from GSE208654, ‘Epithelial_1’ was subdivided into three distinct clusters, as depicted in **Figure 4F**.

### AUCell and cell communication analysis

**Figure 5:**
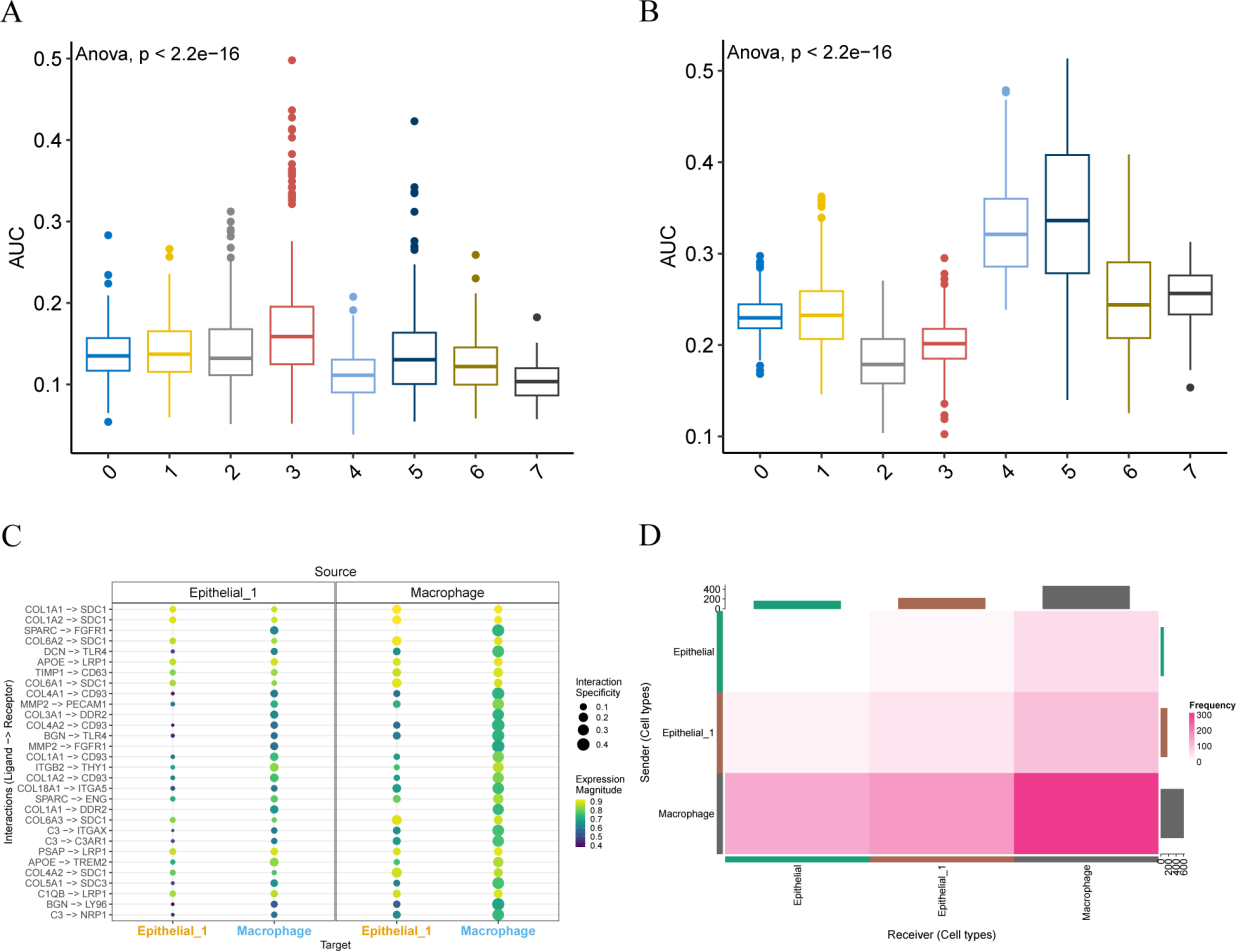
AUCell and cell communication analysis. **(A, B)**. Group comparison plots of AUC scores of epithelial subpopulation 1 marker genes and macrophage marker genes between different cell clusters are visualized. **(C)**. Consensual-based bubble plot of cell communication analysis implemented by LIANA, where the size of the dots represents the confidence level and the color from dark to light represents the stronger communication effect. **(D)**. Heat map of the number of interaction relationship pairs among the three cell types. The deeper the red color, said interaction ligand - receptor on the more the number.

We used the R package AUCell to score the expression of epithelial subpopulation 1 marker genes (**Figure 5A**) and macrophage marker genes (**Figure 5B**) in the idle dataset, visualizing them in the group comparison plots for the single-cell dataset GSE168652 described above. The results showed that the epithelial subpopulation 1 marker genes had the highest area under the curve (AUC) scores in cell subpopulation 3, which aligns with the previously obtained results. Similarly, the macrophage marker genes displayed the highest AUC scores in cell subsets 4 and 5, consistent with our annotation results.

The liana package was employed for consensus cell communication analysis based on different methods. We visualized the intensity (**Figure 5C**) and quantity (**Figure 5D**) of cell communication through bubble charts and heat maps, respectively. Notably, macrophages and Epithelial_1 exhibit multiple ligand-receptor combinations of interaction modes. Moreover, macrophages and Epithelial_1 have a high number of interactions.

### Susceptibility analysis

We applied the bc4Squares function in the Beyondcell package to summarize the drug ranking between macrophages and epithelial subpopulation 1 (**Supplementary Figures S5A, B**). The upper left and lower right corners of the figure contain all selected drugs with low and high sensitivity in the cells. In panels C–E, we specifically focus on triptolide and memantine, two compounds that demonstrate distinct differential effects between macrophages and epithelial cell subpopulation 1. Specifically, epithelial cell subpopulation 1 demonstrated sensitivity to triptolide, while macrophages were more sensitive to memantine.

### Protein expression of macrophage-related genes *CD163*, *CD14*, and *CD68*

**Figure 6:**
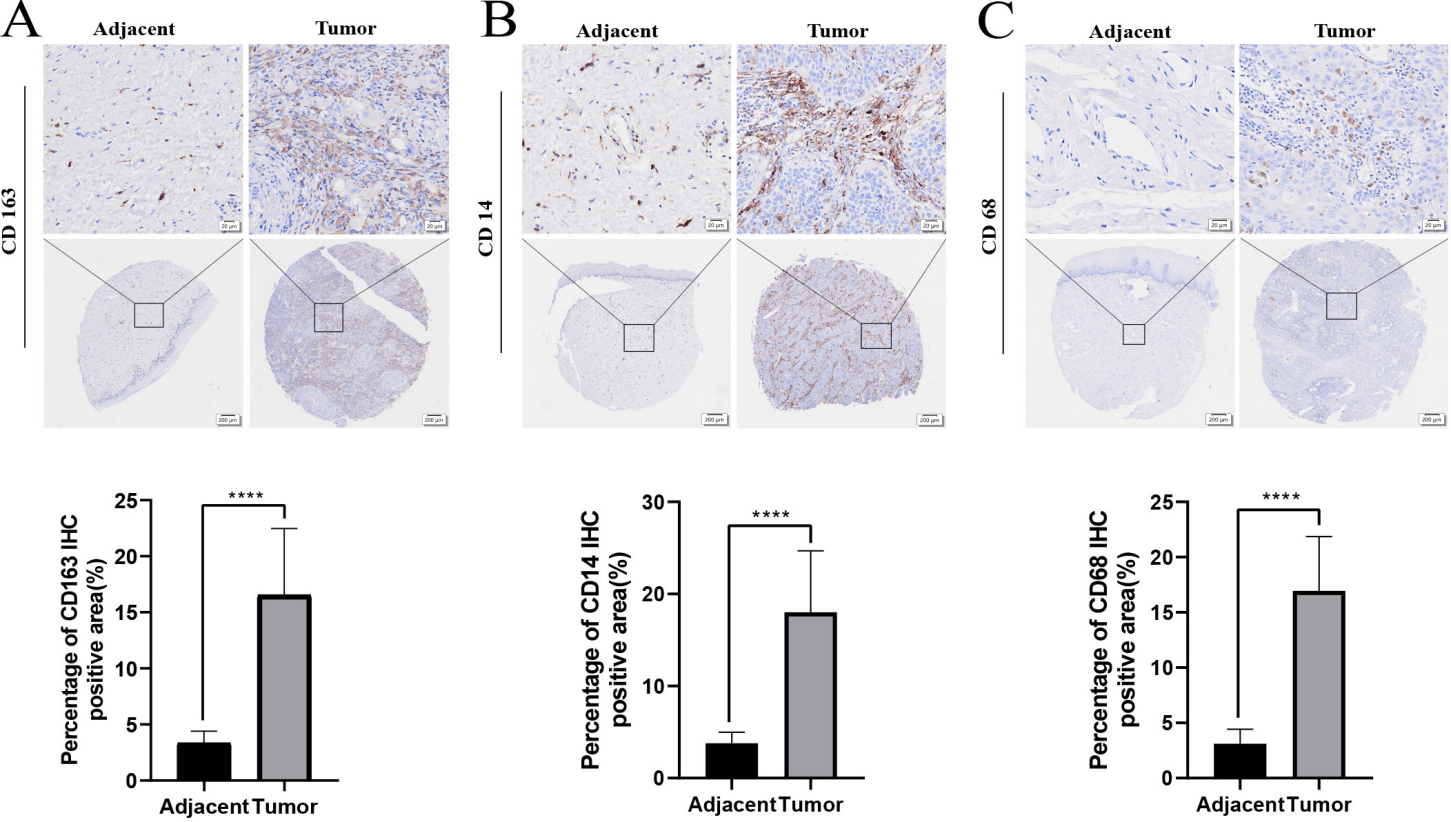
The protein expression of macrophage related genes CD163, CD14, CD68 in adjacent tissues and tumor tissues through IHC **(A-C)**. ****p < 0.0001.

The protein levels of macrophage-related genes, including CD163, CD14, and CD68, were assessed using immunohistochemistry (IHC) on tumor tissues and adjacent tissue microarray. The results revealed a significant increase in the protein levels of CD163, CD14, and CD68 in the tumor tissue compared to adjacent tissue (*p* < 0.0001, **Figures 6A–C**).

## Discussion

CC is one of the most common gynecological malignancies with high morbidity and mortality (30–32). Currently, the treatment of CC includes surgery, radiotherapy, and chemotherapy; however, the efficacy in patients with recurrent and advanced stages remains limited, often resulting in poor prognosis (33–35). For patients progressing after initial treatment, options are sparse, with low response rates to second-line and subsequent chemotherapy, and a median progression-free survival of approximately 3–6 months. Hence, immunotherapy emerges as a promising alternative, particularly as a second-line or subsequent therapy (36). However, the efficacy of immunotherapy varies among patients, with some showing no response, and the underlying mechanisms remain unclear. Therefore, understanding immune escape mechanisms and developing additional immunotherapy strategies is imperative.

Notably, the TME likely plays a vital role in immune escape (37, 38). Additionally, TME significantly influences cancer progression, with diverse signals impacting tumor promotion and suppression (39). HPV infection contribute to immune evasion (40); however, the mechanisms of virus-TME interactions and cancer induction warrant further exploration (19).

The diverse immune cells, such as the cancer-associated fibroblasts (CAFs), endothelial cells, and extracellular matrix (ECM), along with additional tissue-resident cells, contribute to the evolving TME during all stages of cancer (41). Single-cell RNA sequencing (scRNA-seq) reveals the features of cellular gene expression that cannot be observed in bulk RNA sequencing (42). Moreover, it exhibits the crosstalk among tumor cells, immune cells, and CAFs in the TME (43, 44). Therefore, scRNA-seq has many applications in cancers (45–47).

Recent studies have provided valuable insights into the cellular dynamics of the TME in CC. Guo et al. (19) demonstrated that the overall abundance of CD8+ T cells is elevated in cancer tissues, with a notable increase in exhausted T cells (Tex). Mucosal-associated invariant T (MAIT) cells were found to be predominant in high-grade squamous intraepithelial lesions (HSIL), while terminal effector memory RA+ (TemRA) T cells exhibited a biphasic response, initially increasing and subsequently decreasing following HPV infection. Among CD4+ T cell subsets, Th17 cells displayed a similar biphasic trend, whereas Th1-like and regulatory T cells (Tregs) showed progressive infiltration during disease progression. In the myeloid compartment, plasmacytoid dendritic cells (pDCs) accumulated in cancer tissues but exhibited functional impairment, and macrophages underwent a polarization shift from the M1 to the M2 phenotype. Li et al. (48) focused on non-immune cells within the TME, identifying 22,451 fibroblasts and smooth muscle cells (SMCs) that were further classified into 13 distinct clusters based on gene expression profiles. Qu et al. (49) revealed heterogeneous immune cell signaling patterns in the CC TME and identified a subset of cancer-associated fibroblasts (CAFs) that impede lymphocyte infiltration and remodel the extracellular matrix (ECM). Despite these advances, a comprehensive scRNA-seq analysis of epithelial and macrophage dynamics in the CC TME remains lacking. This gap underscores the need for further research to elucidate the intricate interplay between these cell types, which could provide critical insights for developing effective immunotherapeutic strategies.

We analyzed cervical cancer using datasets GSE44001, GSE168652, and GSE208654 for the first time. GSE168652 identified 12 cell clusters, including CD8+ T cells, macrophages, and epithelial cells. CD8+ T cells, despite dysfunction in cancers, are key antitumor agents, with therapies aiming to boost their cancer-fighting abilities. Tumor-associated macrophages (TAMs) have dual roles in cancer, promoting and inhibiting tumor growth. Using scRNA-seq, we detailed macrophage-epithelial interactions in cervical cancer’s tumor microenvironment (TME). Our study integrated multiple analyses to understand TME dynamics, revealing macrophages’ active role and epithelial cells’ lower activity. GO and KEGG analyses linked cell types to specific biological functions and pathways in cervical cancer. Macrophage markers correlated with better survival outcomes, suggesting their potential as prognostic biomarkers and therapeutic targets. Immunohistochemistry confirmed higher macrophage marker expression in cancer tissues, underscoring their prognostic significance for cervical cancer.

TMB in epithelial cell subgroup 1 differs significantly between the high- and low-score groups. Moreover, a high TMB indicates the potential to carry new antigens, rendering the epithelial subgroup a target for activated immune cells. These observations indicate an emerging biomarker for susceptibility to immune checkpoint inhibitors, as assessed by IHC, which is associated with the response to CTLA-4 and PD-1 inhibition in immunotherapy (50–52). The evaluation of the TIDE algorithm showed marked differences in TIDE immunotherapy scores among CESC patients between the high- and low-rating groups of macrophages, while no statistically significant difference was detected in epithelial cell subgroup 1. This finding suggested that different immune microenvironment states have an impact on immunotherapy reactivity, with macrophages potentially playing a key role in influencing the sensitivity of CC patients to immunotherapy.

There are limited and conflicting reports on the prognostic utility of PIK3CA and the assessment of PIK3CA in cervical cancer before radical hysterectomy may help identify patients at higher risk of node-positive disease (53). Maftools mutation analysis revealed that PIK3CA had a higher mutation frequency in both macrophages and epithelial subset 1. PIK3CA in our study, which is a common oncogene, exhibited a high mutation frequency in various cancers (54), and its mutations may have a critical impact on tumor progression and treatment response. One of the biggest obstacles to achieving a long-lasting response to cancer therapies is drug resistance (55). Gabriele Romano et al. found that the mutations PIK3CA E545K and NRAS Q61 are sufficient to generate resistance (56). Combined with our study, by highlighting the presence of PIK3CA mutations in macrophages and epithelial cells and their potential role in tumor progression and drug response, this research suggests avenues for the development of targeted therapies as well as the possibility of using PIK3CA mutation status to guide treatment decisions, which may be possible leads to more effective treatment of CC.

The spatial transcriptome dataset GSE208654 revealed the location of two major spatial locations of macrophages and epithelial cells. It validated the observations of the single-cell dataset GSE168652 through model gene expression levels. Spatial transcriptomic analyses offer insights into the spatial distribution and interactions of various cell types within the TME, thereby enhancing our understanding of tumor heterogeneity and the complexity of its microenvironment.

Finally, we identified potential targeted treatment drugs, triptolide and memantine. Triptolide is a diterpenoid compound isolated from the Chinese herb *Tripterygium wilfordii*. It is effective in treating various autoimmune diseases (57)) and has antitumor properties (58–60). Some studies have shown that triptolide induces protective autophagy in human CC cells, inhibits cell viability, and promotes cell apoptosis by activating targeted autophagy pathways (61). The antitumor effect of triptolide aligns with our conclusion. Memantine is an uncompetitive antagonist with moderate affinity for NMDA (N-methyl-D-aspartate) receptors, primarily used for the treatment of Alzheimer’s and cardiovascular diseases and cancer (62). However, its application in CC has not been reported. It has been reported that triptolide causes apoptosis (63). The development of novel therapeutics is facilitated by the introduction of triptolide and memantine as potential treatments, based on their effects on cellular mechanisms relevant to CC. The creative part is not just finding new medications, but also explaining how they specifically act on various cell types in the TME, leading to more planned, focused, and efficient therapies.

This study has several limitations that warrant consideration. First, while Kaplan-Meier survival analysis identified significant correlations between specific cell marker genes, particularly macrophage marker genes, and both overall survival (OS) and disease-free survival (DFS) in patients, we acknowledge the importance of investigating the co-expression patterns of macrophage and epithelial marker genes to better elucidate cell-cell interactions. Due to constraints in time and resources, these analyses were not included in the current study but are planned for future investigations to enhance the comprehensiveness of our findings. Second, although we utilized publicly available datasets and validated the expression of macrophage markers in cervical cancer (CC) and normal tissues using immunohistochemistry (IHC), the underlying biological mechanisms driving these interactions remain to be fully elucidated. Further validation through *in vivo* and *in vitro* functional assays, as well as larger-scale clinical studies, will be essential to substantiate our observations. Third, research on the prognosis and therapeutic outcomes of patients with refractory cervical cancer, including recurrent, persistent, or metastatic disease, remains limited. Future studies should focus on exploring the associations between distinct disease subgroups and potential therapeutic strategies to address this unmet clinical need.

In conclusion, this study emphasizes the necessity and promise of personalized medicine in the treatment of cancer by providing a thorough understanding of the cellular and genetic makeup of the TME, with a focus on the function of TAMs and the consequences of particular genetic mutations. This implies that a better understanding of the TME at the molecular and cellular levels may influence treatment choices and improve patient outcomes in the long run. In order to provide a clear roadmap for the continued development of CC treatment, we suggest specific areas for future research, such as investigating the role played by macrophages in the progression of cancer, the effects of triptolide and memantine on macrophages and epithelial cells, and the interaction between drug therapy and specific genetic mutations. This study lays the groundwork for important advancements in the diagnosis, prognosis, and treatment of cervical cancer by creatively focusing on the details of macrophage roles, the effects of TAM and epithelial cell mutations, and investigating novel drug therapies based on TME dynamics. Clinical ramifications could include improved patient outcomes and increased survival rates in CC, as well as the creation of tailored, targeted treatment plans and more accurate prognostic evaluations.

## Data Availability

Publicly available datasets were analyzed in this study. The datasets generated and analyzed during the current study are available in the Gene Expression Omnibus (GEO) (https://www.ncbi.nlm.nih.gov/geo) and The Cancer Genome Atlas (TCGA) (https://portal.gdc.cancer.gov/).
