# Supplementary material for: Epithelial and macrophage cell interaction in cervical cancer through single-cell RNA-sequencing and spatial analysis
Source: Front Immunol. 2025 Apr 9;16:1537785. doi: 10.3389/fimmu.2025.1537785 (PMC12014682; doi:10.3389/fimmu.2025.1537785)
Supplement: Supplementary file 1 [file DataSheet1.docx]

Supplementary Material

# Supplementary Figures and Tables

## Supplementary Figures

**
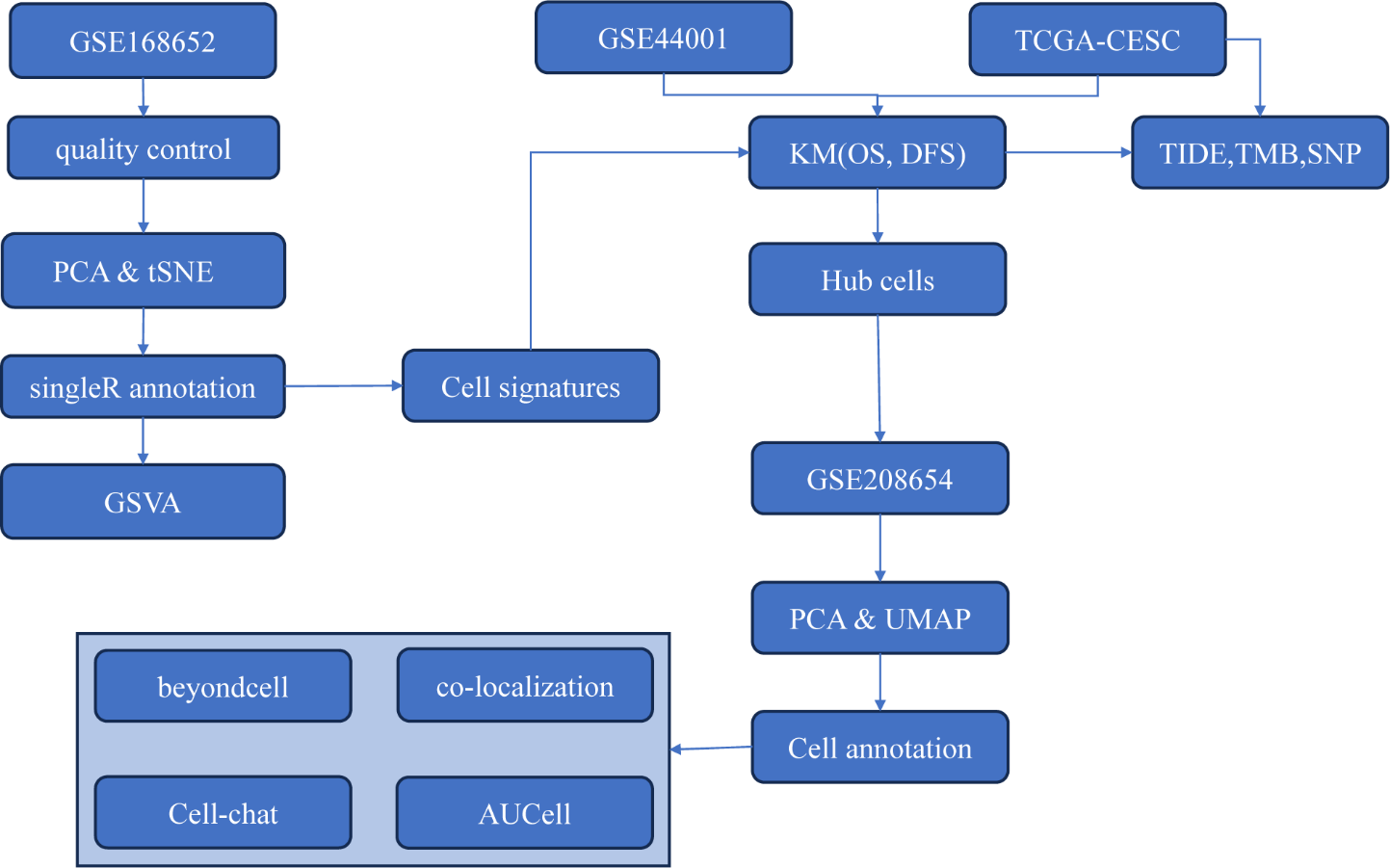
**

**Supplementary Figure 1.** Flow diagram of single cell spatial transcriptome. PCA, Principal Component Analysis; tSNE, t-Distributed Stochastic Neighbor Embedding; GSVA, Gene Set Variation Analysis; KM, Kaplan-Meier; OS, Overall survival; DFS, Disease-free survival; TIDE, Tumor Immune Dysfunction and Exclusion; TMB, tumor mutation burden; SNP, single nucleotide polymorphism; UMAP, Uniform Manifold Approximation and Projection; AUC, Area Under the Curve.


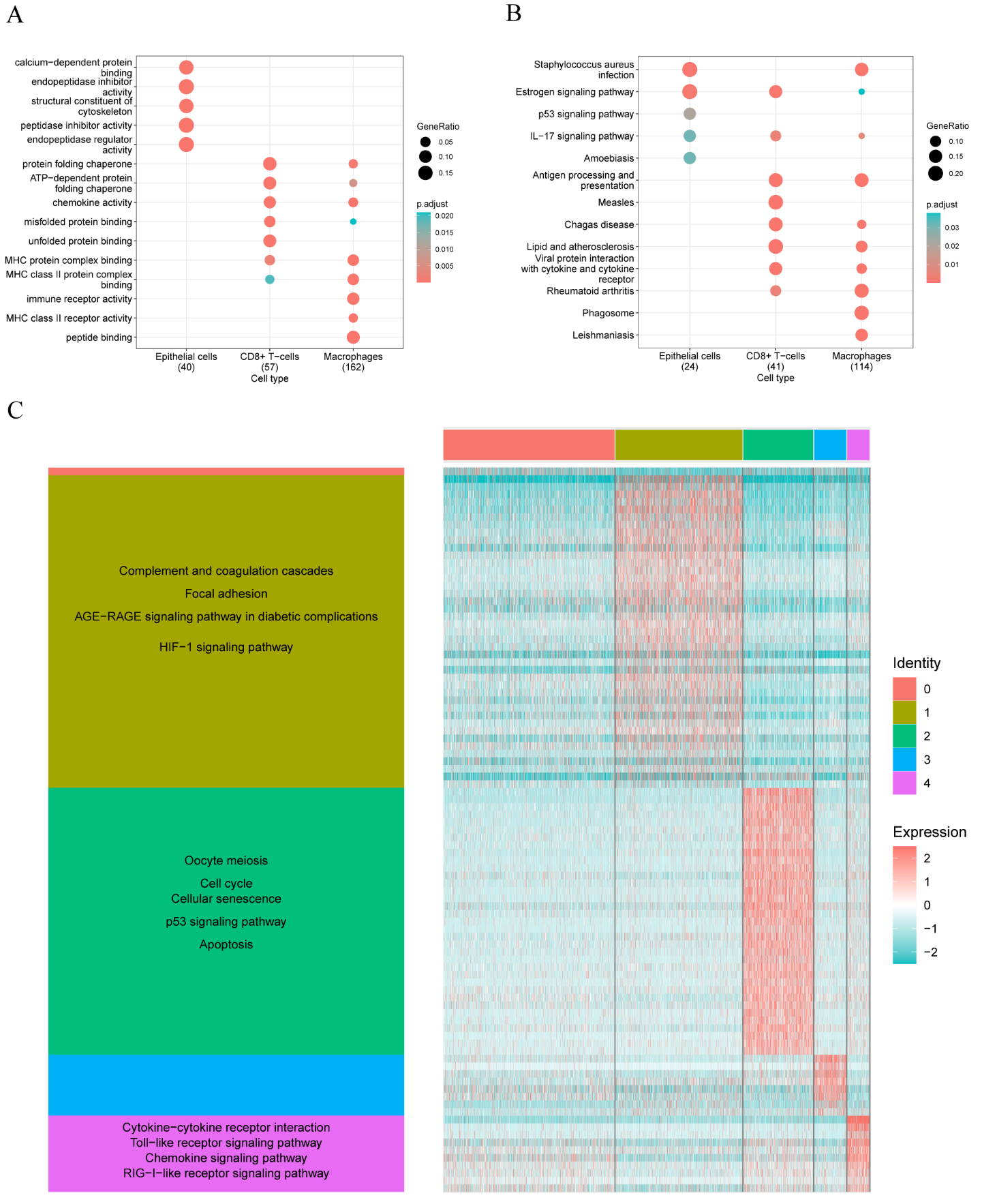


**Supplementary Figure 2.** Gene set enrichment analysis. (A). Bubble plot display of gene ontology (GO) enrichment analysis results of different cell marker genes. (B). Bubble diagram display of KEGG pathway enrichment analysis results of different cell marker genes. (C). Heat map of marker genes and pathway (KEGG) enrichment analysis results of different subsets of epithelial cells.


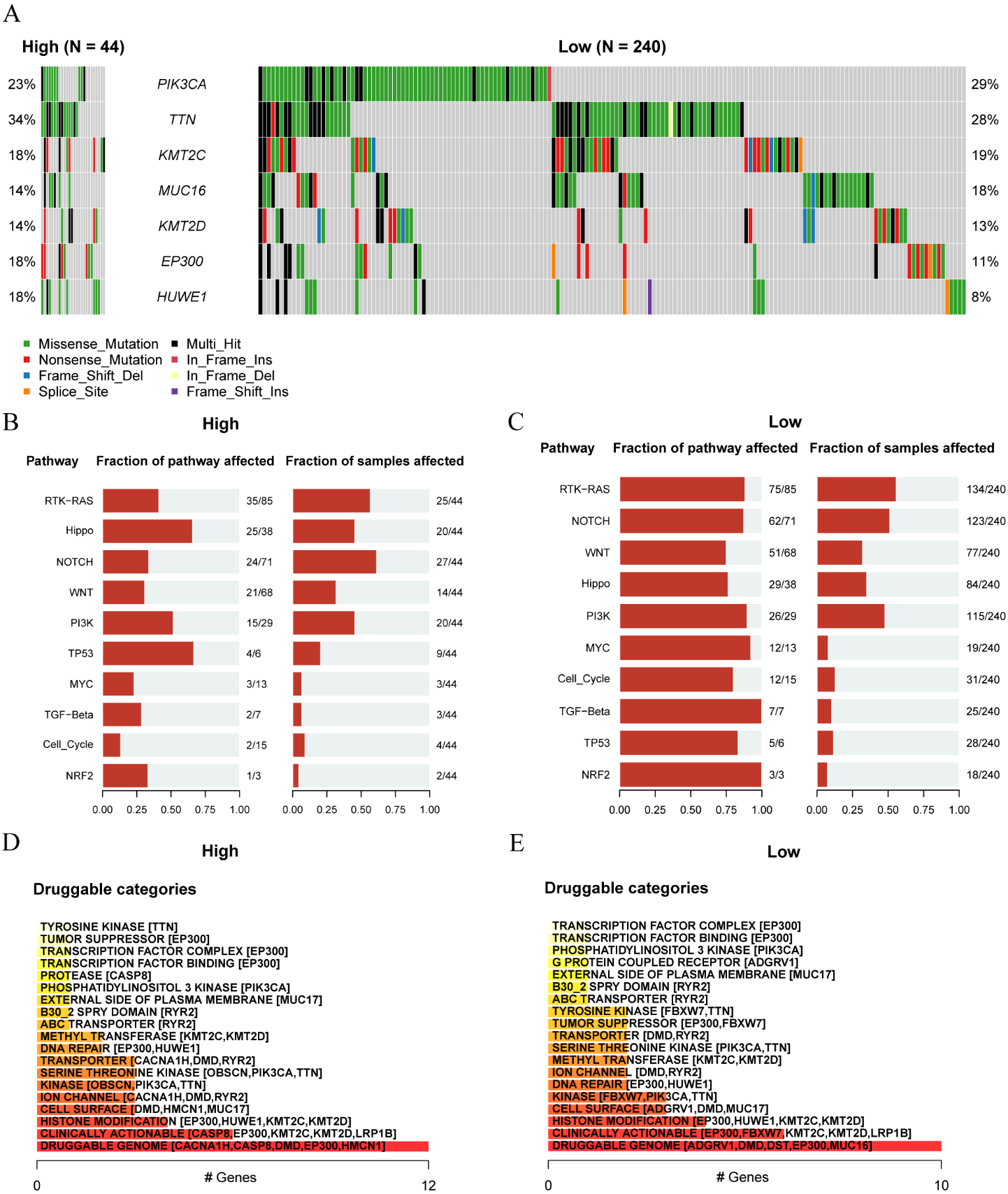


**Supplementary Figure 3**. Somatic mutation analysis of high and low macrophage groups. (A). Mutant gene landscape map of TCGA-CESC macrophage high and low groups. (B). Analysis of biological functions affected by mutations in patients with TCGA-CESC macrophage high grouping. (C). TCGA-CESC macrophages affected by mutations in patients with low biological function analysis. (D). Classification of potentially actionable genes in the TCGA-CESC macrophage high group. (E). TCGA-CESC macrophages in low potential might be classified medicine gene. Behind each classification in brackets are in the TOP five genes, abscissa for medicine gene in the classification number.


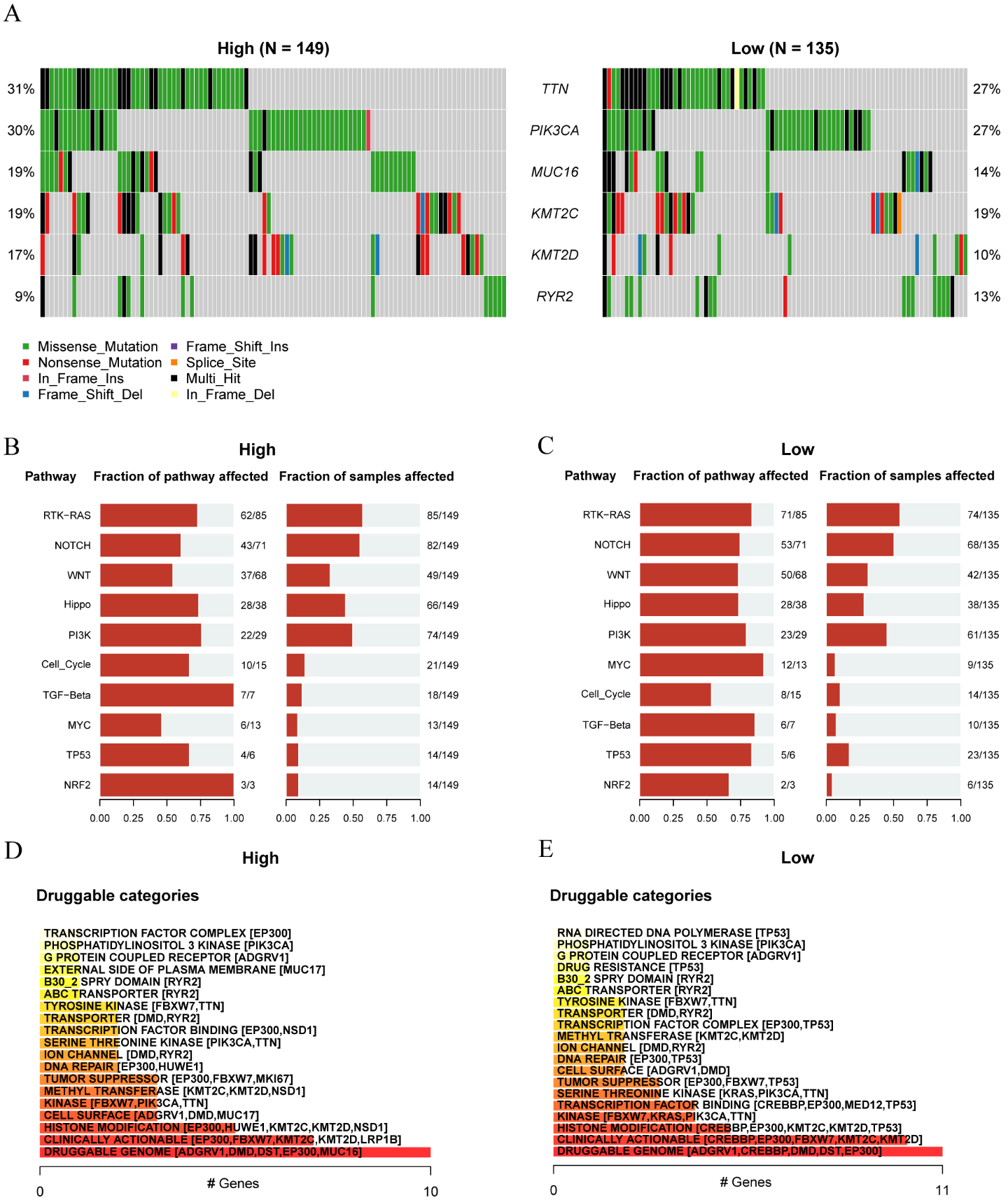


**Supplementary Figure 4**. Somatic mutation analysis of high and low groups in epithelial subpopulation. (A). Mutant gene landscape map of epithelial subpopulation 1 high and low groups in TCGA-CESC. (B). Analysis of biological functions affected by mutations in patients in TCGA-CESC epithelial subpopulation 1 high group. (C). Analysis of biological functions affected by mutations in patients in TCGA-CESC epithelial subpopulation 1 low group. (D). Classification of potentially actionable genes in the TCGA-CESC epithelial subpopulation 1 high group. (E). Classification of potentially actionable genes in the TCGA-CESC epithelial subpopulation 1 low subgroup. Following each category are the TOP 5 genes in parentheses, and the abscissus is the number of genes in the category of drugable genes.


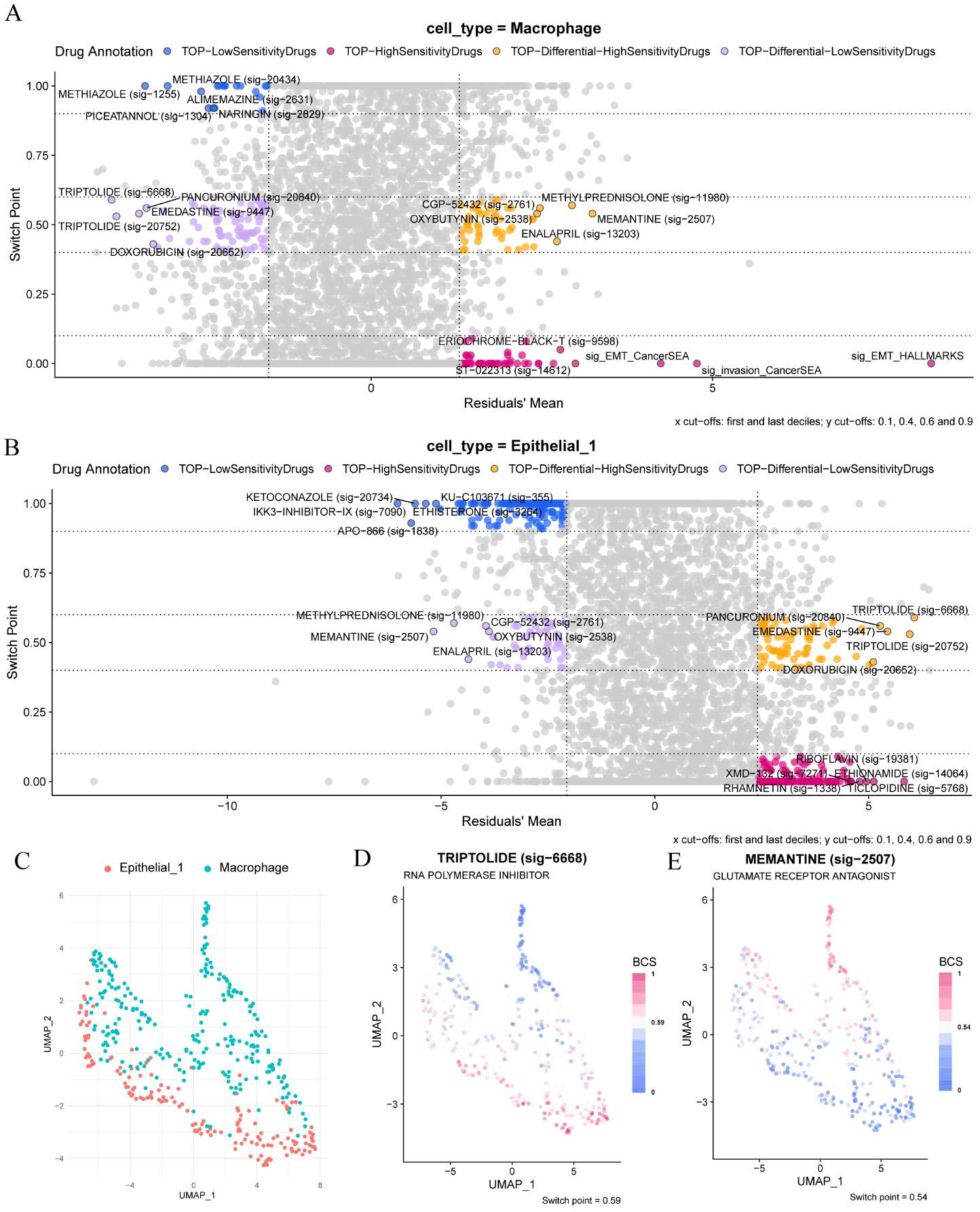


**Supplementary Figure 5.** Susceptibility analysis. (A-B). Summary plot of drug susceptibility ranking in macrophage and epithelial subpopulation 1. (C-E). sensitivity of different drugs sensitivity in the scatter plot in two kinds of cells.

**1.2 Supplementary Table**

**Supplementary Table 1. Data information.**

|  | **TCGA-CESC** | **GSE44001** | **GSE168652** | **GSE208654** |
| --- | --- | --- | --- | --- |
| **Platform** | TCGA | GPL14951 | GPL24676 | GPL24676 |
| **Species** | Homo sapiens | Homo sapiens | Homo sapiens | Homo sapiens |
| **Tissue** | cervical cancer | cervical cancer | cervical cancer | cervical tissue |
| **Samples in CESC Group** | 304 | 300 | 1 | 1 |
